# Wanted: Annual Subscriptions

**Published:** 1906-06-16

**Authors:** A. William West

**Affiliations:** Treasurer of St. George's- Hospital, S.W.


### WANTED: ANNUAL SUBSCRIPTIONS.

Mr. A. William West, Treasurer of St. George's Hospital, S.W., writes under date June 7th, 1906, as follows:—

Your heading "Wanted: Annual Subscriptions" in your issue of the 2nd inst. is perfectly correct, but I should like just to point out that in 1901 a special effort to obtain further support for St. George's Hospital was made, with the result that the annual subscriptions increased from £5,441 5s. in 1901 to £6,547 13s. 9d. in 1902. It is true that in 1905 this amount had fallen to £6,160 8s. 6d., a fact much to be regretted, and any new annual subscriptions will be gratefully received.

. ·. Why not return then to the successful plan of 1901 and continue it each year as we suggested?—Ed. The Hospital.

